# Controlling neural network responsiveness: tradeoffs and constraints

**DOI:** 10.3389/fneng.2014.00011

**Published:** 2014-04-29

**Authors:** Hanna Keren, Shimon Marom

**Affiliations:** ^1^Network Biology Research Laboratory, Faculty of Electrical Engineering, Technion - Israel Institute of TechnologyHaifa, Israel; ^2^Department of Physiology, Faculty of Medicine, Technion - Israel Institute of TechnologyHaifa, Israel

**Keywords:** neural network, control, closed loop, stability, electrophysiology, response fluctuations, multi-electrode array

## Abstract

In recent years much effort is invested in means to control neural population responses at the whole brain level, within the context of developing advanced medical applications. The tradeoffs and constraints involved, however, remain elusive due to obvious complications entailed by studying whole brain dynamics. Here, we present effective control of response features (probability and latency) of cortical networks *in vitro* over many hours, and offer this approach as an experimental toy for studying controllability of neural networks in the wider context. Exercising this approach we show that enforcement of stable high activity rates by means of closed loop control may enhance alteration of underlying global input–output relations and activity dependent dispersion of neuronal pair-wise correlations across the network.

## 1. Introduction

The responsiveness of neural networks to repeated stimuli is inherently variable. This variability is expressed in broadly-distributed statistics of response features, reflecting a rich repertoire of cellular level processes, covering practically every observable timescale (Arieli et al., 1996; Marder and Goaillard, 2006; Faisal et al., 2008; Marom, 2010). From the medical applicative point of view, network response variation poses a challenge, motivating initiatives to devise closed loop schemes intended for the whole brain level (DiLorenzo, 2002; Rolston et al., 2009; Lee et al., 2010; Rosin et al., 2011; Berenyi et al., 2012). As this approach is potentially useful, the underlying tradeoffs and constraints involved remain a matter for trial-and-error exploration; this is due to the obvious complications entailed by studying whole brain dynamics.

In this work, we take advantage of a reduced *in vitro* preparation, combined with closed loop stimulation algorithms, in order to (1) demonstrate the feasibility of controlling network response features over extended time scales and (2) expose impacts of such control on underlying network properties. The said reduced preparation is a large-scale cortical network developing *in vitro*, on top of a substrate integrated multi-electrode array (MEA). This preparation proved useful, over the past 10–15 years, as a toy model in the study of functional network processes, ranging from development and adaptation to learning and stimulus representation (Maeda et al., 1995; Kamioka et al., 1996; Jimbo et al., 1998, 1999; Tateno and Jimbo, 1999; Shahaf and Marom, 2001; Corner et al., 2002; Eytan et al., 2003; Shahaf et al., 2008). A recent series of studies demonstrated the efficacy of several closed loop applications in controlling aspects of activity in these *in vitro* large-scale cortical networks (e.g., Wagenaar and Potter, 2004; Wagenaar et al., 2005; Arsiero et al., 2007; Rolston et al., 2010; Wallach et al., 2011; Weihberger et al., 2013). Here we implement one of these approaches, the so-called “response-clamp” procedure: a PI (Proportional-Integral) negative feedback algorithm (Wallach et al., 2011; Wallach, 2013), in order to control response features *in vitro*. In the present context we define controllability as a capacity to quench variations of functionally-relevant response features. We show that the response-clamp procedure may easily control the probability as well as the latency of network responses to repeated stimuli. At the same time we find that such control, by the very fact of enforcing relatively high activity levels, may lead to compromised stability of other network features. This latter point is demonstrated by monitoring network input–output relations as well as pair-wise correlations between latencies to first spikes.

## 2. Results

**Figure 1:**
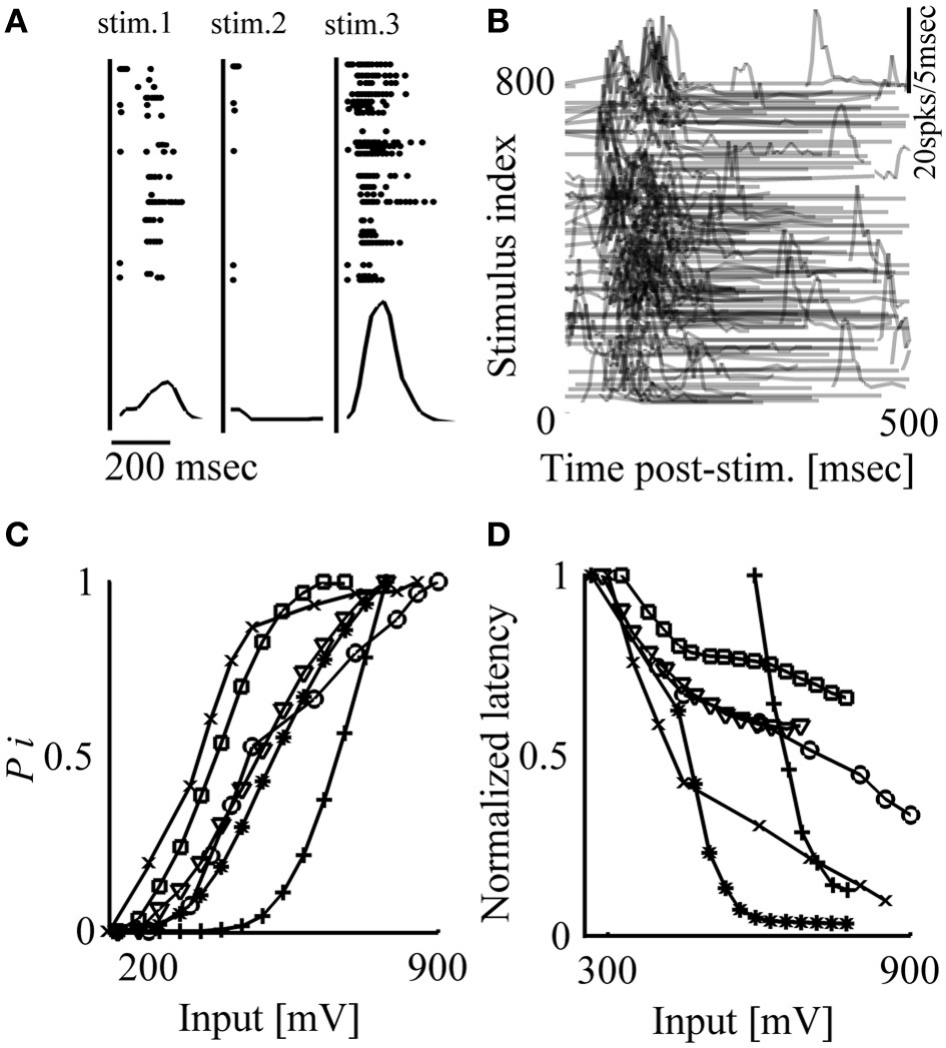
**Network response is highly variable; response probability and latency are correlated to stimulation amplitude. (A)** Top: Responses to three consecutive stimuli (300 mV, 0.2 Hz). Stimulation events are depicted by vertical lines; points represent spikes detected by different electrodes (vertical axes). Bottom: Population post-stimulus time histograms (pPSTH) of all detected spikes, binned to 5 ms. **(B)** Responses to 800 stimuli (300 mV, 0.2 Hz). Each row represents response to one stimulus delivered at time zero; for clarity, only values larger than zero activity are depicted. **(C, D)** Response probability and latency are monotonically affected by stimulation amplitude; stimuli (from 100 to 900 mV; 50 mV bins) are delivered at 0.2 Hz in a randomized order. Each presented value of response probability and latency was calculated from at least 10 responses. Results extracted from six different networks are shown.

When an *in vitro* network of cortical neurons is stimulated locally through the multi-electrode array, a synchronous burst of spikes can be evoked, spreading across extended parts of the network (Maeda et al., 1995; Pinato et al., 1999; Jimbo et al., 2000; Wagenaar et al., 2004). Following the notation used in a previous study (Eytan and Marom, 2006), we denote this threshold-governed phenomenon a Network Spike (NS). When periodically delivered, these stimuli give rise to fluctuations in both the occurrence probability and the temporal envelope (latency, duration, amplitude) of evoked NSs. This is demonstrated in Figure 1A, where three consecutive responses are shown, extracted from a long series of responses to repeated identical voltage stimuli delivered at a frequency of 0.2 Hz. Variations are observed in the identity of participating neurons (as reflected in the identity of active electrodes), in the number of spikes detected by each electrode in response to stimuli, as well as in the latencies of emitted spikes. These variations are also apparent at the level of population responses, NSs, as shown in the lower panels of Figure 1A. Figure 1B shows NSs evoked by a series of 800 such stimuli, revealing slowly varying network response features, extending over many minutes.

A basic requirement, before control of the variations shown above may be considered, is that there be consistent relations between input parameters (e.g., stimulus shape, amplitude, duration, frequency) and the feature to be controlled. We chose to focus our attention on the effects of stimulus amplitude, previously shown to monotonically impact on various response features *in vivo* (e.g., Day et al., 1989; Walker et al., 2012; Park et al., 2013). In the present *in vitro* setting, the stimulus is a square 200 μs voltage pulse, the amplitude of which may vary from 100 to 1000 mV, constrained from above by the electrochemistry of electrodes involved. Figures 1C,D show monotonic relations between stimulus amplitude and two key response features—occurrence probability and latency of evoked NSs. Conveniently, in most networks the range of stimulus amplitudes needed in order to modify NS latency and probability is found to be within the above mentioned (electrochemically imposed) boundary; such are the networks used in the present study. However, it is instructive to note that in cases where NS responsiveness is relatively low and cannot be explored by the above mentioned range of stimulation amplitudes, the matter may be rectified by pharmacologically blocking inhibitory synapses.

Given the consistent monotonic relations between stimulus amplitude, NS response probability and latency, a proportional-integral (PI) clamp circuit may be implemented. A response feature *y*(*n*), be it the occurrence of a NS (a binary value) or NS latency (a positive, continuous value), is recorded following each stimulus *u*(*n*). The PI control algorithm adjusts the amplitude of the subsequent stimulus *u*(*n* + 1) based on the “error” *e*(*n*)—the difference between the response output and the desired value of response feature, y^*^(*n*). In the case of response probability, the output is an estimated value, denoted *P*_τ_, and the desired value is accordingly denoted P^*^. Further technical details are described in Materials and Methods as well as in Wallach et al. (2011); Wallach (2013).

**Figure 2:**
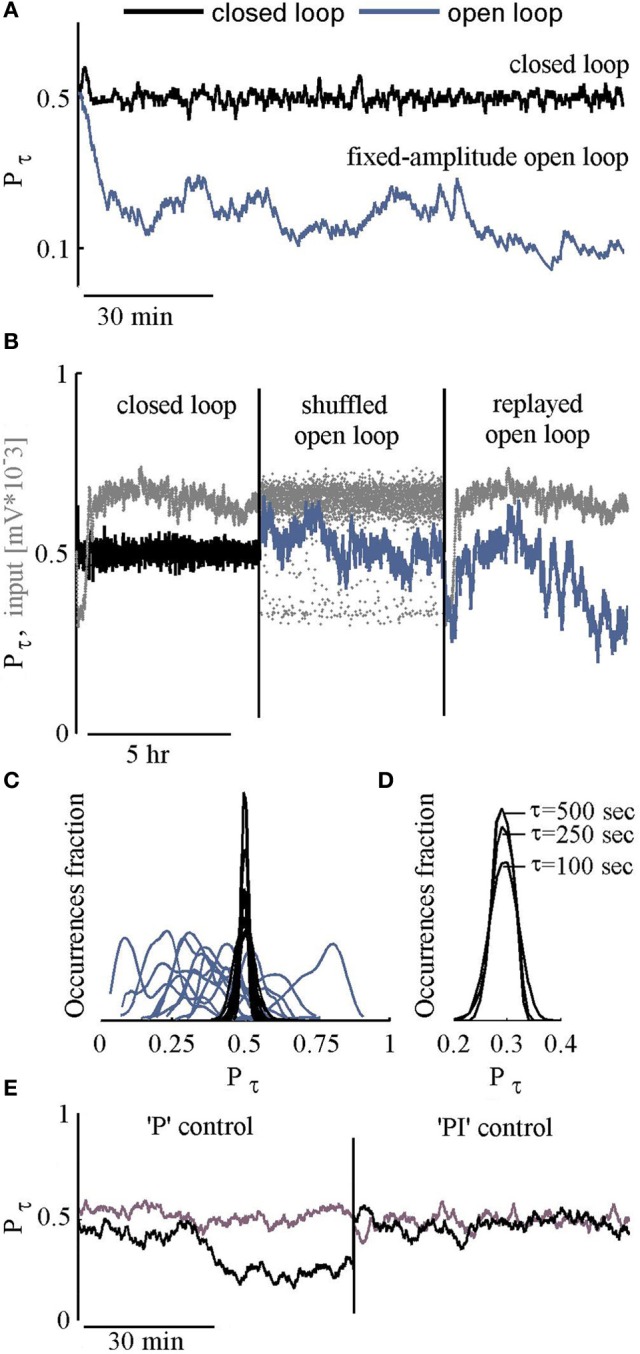
**Feasibility of control. (A)** Response probability values under closed loop control, targeted to P^*^ = 0.5 are depicted black. The blue line shows the network response probability to a series of constant amplitude stimulation (the mean of above closed loop amplitudes). **(B)** Three consecutive sessions: Closed loop followed by open loop sessions where the input is a shuffled or a replayed version of the closed loop series of inputs (stimulation amplitudes). Input time series are depicted Gray. **(C)** Histograms of response probabilities under closed (Black) and open (Blue) loop conditions. **(D)** Histograms of response probabilities under closed loop stimulation, targeted to P^*^ = 0.3, using different τ values (500, 250, or 100 s). **(E)** Response probability of two networks under closed loop using only the “P” component of the controller, followed by closed loop with both “P” and “I” components (see Materials and Methods for specific values).

The efficacy of the PI controller in clamping the response probability of our large scale networks is exemplified in the black trace of Figure 2A. The desired responsiveness (P^*^) was set to 0.5 (50% probability of a response to stimulation). The response to each stimulus is defined as a binary value. A detected NS within a post-stimulus time window of 10–800 ms, is designated 1; in the absence of a NS in that time window, the response is designated 0. Response probability *P*_τ_, is computed by averaging the resulting binary time series, using an exponential kernel with a characteristic time scale τ = 250 s. For comparison, the blue trace (Figure 2A) shows *P*_τ_ in an open loop condition, where a series of stimuli at a constant amplitude (300 mV, the average of the closed loop input series) is delivered.

A comparison of closed loop results to a constant amplitude open loop stimulation, as demonstrated in Figure 2A, is not sufficiently convincing. What one might wish to see is a comparison to varying open loop series. Two such open loop series are considered: (1) replayed and (2) shuffled versions of the stimulation series generated by the controller in the closed loop setting. Figure 2B shows the resulting fluctuations of response probability in both types of open loop conditions (blue), compared to the closed loop condition (black); the stimulus time series are depicted in gray. Results obtained by long term (3–5 h) of 12 such experiments are summarized in Figure 2C, demonstrating quenched fluctuations of response probability values under closed loop.

The choice to set the controller's time scale (τ) to 250 s reflects a tradeoff between reduction of fluctuations on the one hand, and capacity to modulate target response probabilities over minutes (as demonstrated in a later section), on the other. As shown in Figure 2D, the impacts of a 5-fold change in the value of τ on the standard deviation of response fluctuations are negligible. Related to this issue, given the fact that integration time of 500 s implies 100 stimuli, we suspect that the observed fluctuations under closed loop control, scratch the limits of our system. Obviously, the gains of the integral and proportional components are crucial here. But beyond these, the origin of this limit—biological (e.g., interference by spontaneous sporadic activity, some sub-threshold dynamics, etc.) or technical (e.g., electrodes chemistry, setup movements, etc.)—remains open at this stage. And, finally, note (in Figure 2E) that both the integral and proportional components of the controller are needed; while use of the proportional component only might lead to quenching of response variability, the resulting response probability may not be effectively controlled.

**Figure 3:**
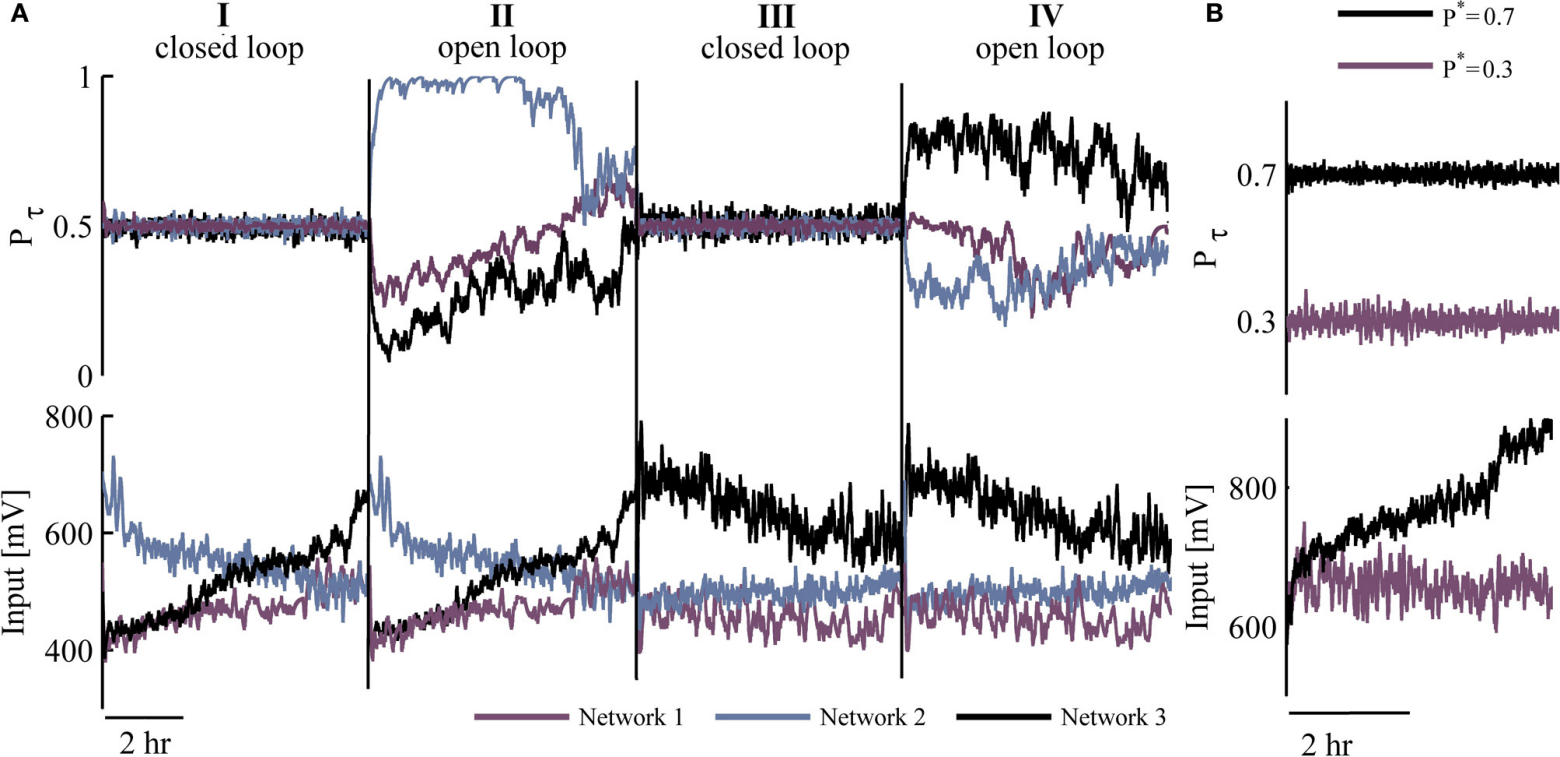
**Efficacy and robustness of control. (A)** Top: Two sessions (I and III) of controlling response probability to P^*^ = 0.5, interleaved by sessions (II and IV) of open loop replay stimulation. These are shown for three different networks (colored). Bottom: Stimulation amplitudes corresponding to top traces. **(B)** A single network is clamped to either P^*^ = 0.3 (purple) or P^*^ = 0.7 (black) response probability; upper panel presents response probability values whereas inputs (stimulation amplitudes) are presented in respective colors at the bottom panel.

Figure 3A depicts three different networks that were exposed to two closed loop sessions (I and III, 5 h each), interleaved by open loop replay sessions (II and IV, 5 h each). The results, beyond demonstrating the ability to implement network control over many hours, uncover interesting phenomena observed in all the cases we have studied: (1) Control of different networks to the same desired responsiveness P^*^ entails qualitatively different input series (i.e., generated stimulation amplitude values). (2) Different input time series are required in order to stabilize network responsiveness in repeated closed loop sessions; this is apparent from the input traces of the bottom panel (sessions I and III).

The target value of response probability (P^*^) is not limited to being 0.5. Figure 3B demonstrates implementation of control at two different desired probabilities (0.7 and 0.3) in the same network. Note the impact of these differences on the input time series (lower panel), reflecting a trend in our observations: When response probability is clamped to higher values, the required stimulation amplitudes are higher on average (across five networks clamped to P^*^ = 0.7, the mean stimulation amplitude was 30% higher compared to clamping to P^*^ = 0.2–0.3). Note that the impact of the value of P^*^ on the quenching of response fluctuations under closed loop control, does not seem to be significant; the standard deviation of *P*_τ_ remains between 0.02 and 0.03 across 16 closed loop sessions to P^*^ ranging from 0.2 to 0.7.

**Figure 4:**
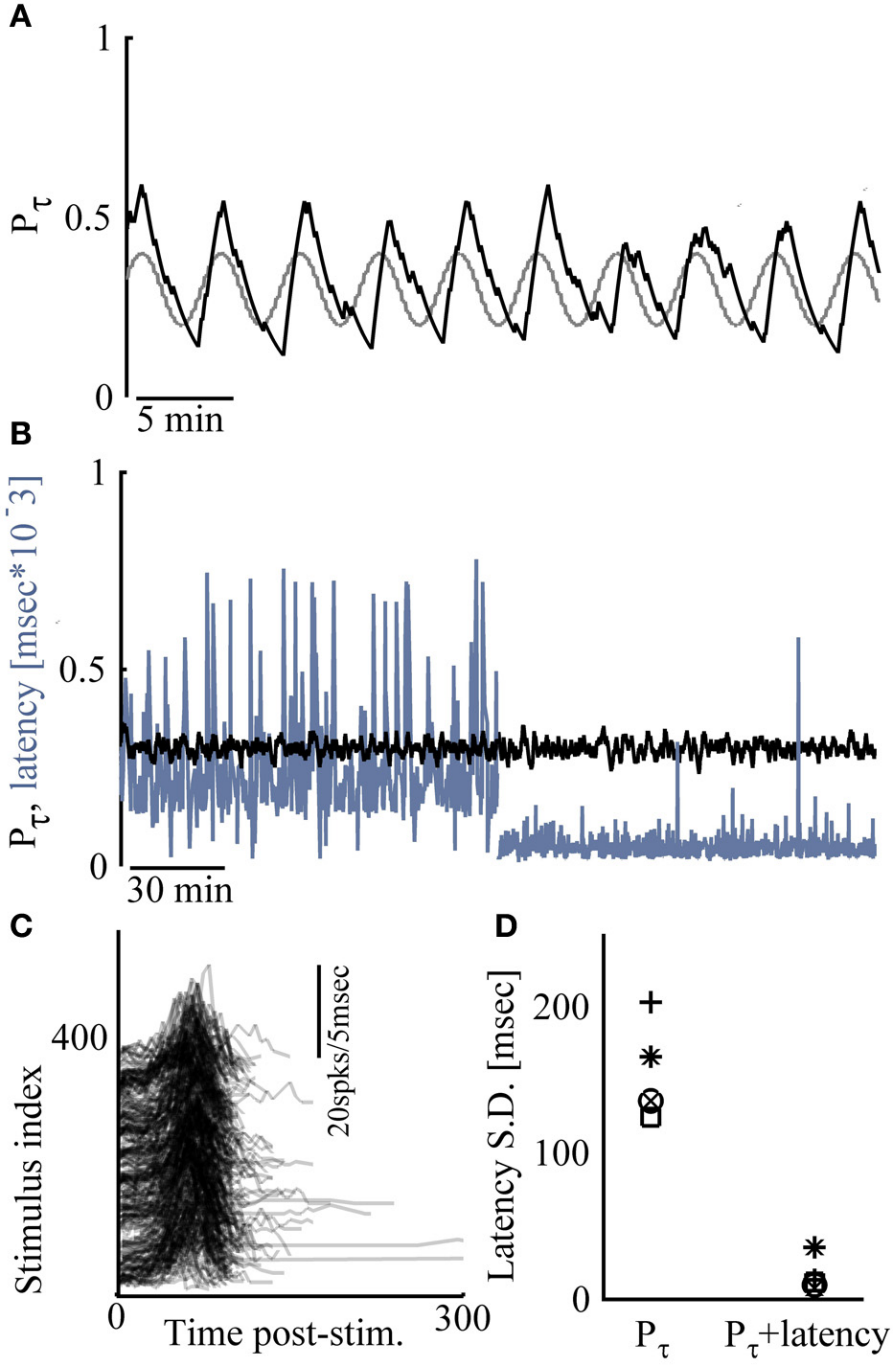
**Extensions of network control. (A)** Response probability is manipulated by sine-wave control; the desired response probability is depicted gray. **(B)** Control of response probability (to P^*^ = 0.3) for the first 2 h, after which control of both response probability *and* latency (to 10–80 ms) is exercised. **(C)** Responses to 400 consecutive stimuli during the simultaneous control (only values above zero activity are depicted). **(D)** Standard deviation of response latency values under response probability control and under simultaneous control is presented for five different networks.

Figure 4 demonstrates extensions of the above described closed loop control capabilities. In Figure 4A an alternating, sine-wave like control of response probability is shown. In such cases, the time scale chosen for computing *P*_τ_ is important, affecting both the phase shifts and amplitudes of output patterns. Another extension involves simultaneous control over two response features: Where the sensitivity of response features to the input are correlated, control of more than one feature may be considered, limited by the nature of the correlation. This is demonstrated in the results of Figures 4B–D, where the nature of correlations between input (stimulation amplitude), response probability and response latency (see Figures 1C,D), is taken advantage of. Naturally, such simultaneous procedure is severely limited if control to relatively high response probability is attempted together with relatively delayed, long latencies to NSs. Figure 4D summarizes five different simultaneous control experiments (in five different networks).

**Figure 5:**
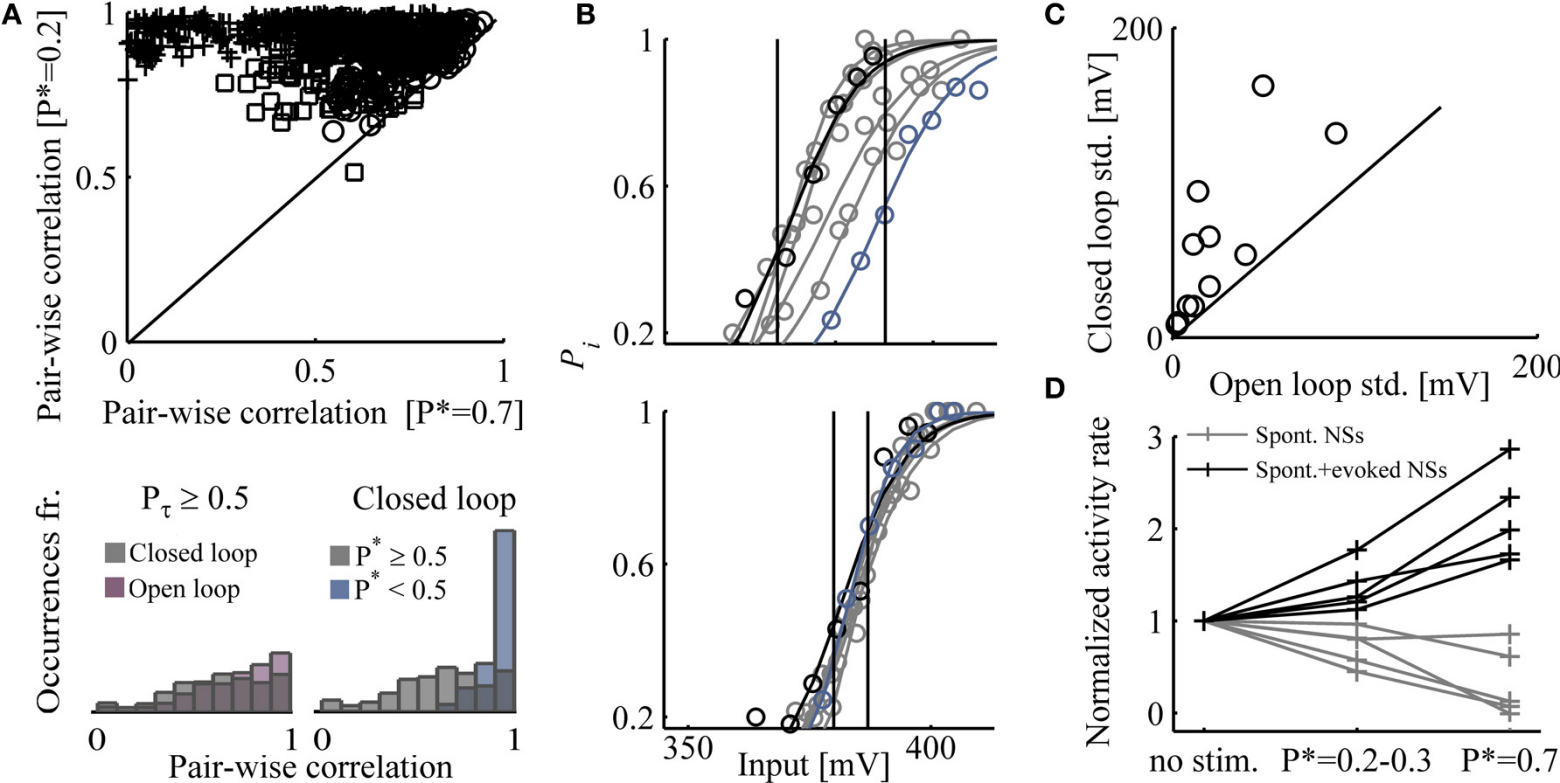
**Impacts of high activity rates enforced by closed loop. (A)** Top: Pair-wise correlation coefficients (Pearson's correlation) between times to first spike, under control (P^*^ = 0.2 and P^*^ = 0.7); results from three networks are shown, each depicted by a different marker. First spikes that their latency values are <10 or >400 ms are discarded. Bottom: Right-hand panel shows that the distribution of pair-wise correlations (from nine different networks) is significantly narrower for P^*^ < 0.5 compared to P^*^ ≥ 0.5. The left-hand panel shows that the distribution of pair-wise correlations (six different networks) at *P*_τ_ ≥ 0.5 is equally broad, whether under closed or open loop conditions. **(B)** Input–output relations calculated over 40 min epochs along 5 h of closed loop control stimulation (upper panel) and 5 h of open loop stimulation (lower panel). Stimulation amplitudes are binned to 5 mV; *P*_*i*_ is calculated for each amplitude bin (a minimum of 10 responses is required for inclusion of a bin). Input–output relations of the first and last 40 min epochs are depicted black and blue, respectively. Continuous lines depict a fit to the Boltzmann equation. The standard deviation of inputs that evoke responses over an arbitrary range of 0.45–0.55 was calculated for both stimulation protocols (11 comparisons in nine networks) and presented in panel **(C)**. **(D)** The rates of spontaneous and evoked NSs under closed loop, at two different desired response probabilities (P^*^ = 0.2–0.3 and P^*^ = 0.7). Rates are normalized to frequency of spontaneous NSs (i.e., no stimuli session).

The above enforcement of response features using closed loop control, entails instabilities at the underlying levels of network activity organization. Figure 5A (top), demonstrates that clamping network responsiveness to relatively high values of P^*^ = 0.7 causes a significant dispersion of pair-wise correlations between latencies to first spikes, detected in the different electrodes, compared to P^*^ = 0.2. A summary of the effects of P^*^ values on the stability of pair-wise correlations is shown in the bottom right panel, where the results from nine networks are integrated; pair-wise correlations between times to first spikes are broadly-distributed when higher P^*^ are maintained. To convince ourselves that the dispersion effect is due to higher activity rather than to the closed loop control itself, we extended each closed loop experiment by replaying the controller input series (or a shuffled version of it) in open loop conditions. We found that similar dispersions of correlation values occur under open loop conditions in high *P*_τ_ (see bottom left panel of Figure 5A).

Beyond dispersed pair-wise correlations of times to first spikes, control of network response probability compromises the stability of global, population-level input–output relations. Figure 5B demonstrates this effect in the case of one network, where the relations between stimulation amplitude and response probability are estimated along a 5 h closed loop control (top) and 5 h shuffled open loop stimulation (bottom). The sessions were sliced to 40 min epochs; for each epoch, the output, response probability, was estimated by calculating the fraction of evoked NSs in response to binned stimulation amplitudes (denoted *P*_*i*_). Note the right-shift of input–output relations in closed loop control (top) compared to the stable input–output in open loop conditions. Of nine networks tested, eight showed such right-shift (i.e., becoming less sensitive to stimulus amplitude) along the closed loop session, whereas only one drifted to the left (i.e., becoming more sensitive). Figure 3 is as well informative in this context.

Quantification of input–output stability is offered in Figure 5C, generated as follows: In the above nine mentioned networks, the response probability (*P*_*i*_) of the input–output relations extended across a range of 0.55 > *P*_*i*_ > 0.45. In these networks, the standard deviation of the input spanning this range was calculated (the extent of such amplitude values is represented by vertical lines in Figure 5B); a presentation of this value for each network in both closed and open loop conditions, is given in Figure 5C. In all cases, input–output stability thus estimated is compromised by the closed loop control. The (mostly rightwards) drift of input–output relations induced by closed loop control, is not fully reversible upon change from the closed loop to the open loop conditions (data not shown).

Finally, Figure 5D shows that there is a tradeoff between evoked and spontaneous NSs. As the rate of responsiveness increases by the closed loop controller, the fraction of spontaneously occurring NSs decreases. This result is congruent with data presented by Wagenaar et al. (2005), where feedback stimulation was implemented in order to increase the total spike rate across the network, causing a decrease in spontaneous NSs.

## 3. Concluding remarks

Cortical networks developing *in vitro* are acknowledged as an experimental platform where features of *in vivo* activity may be effectively analyzed (Droge et al., 1986; Jimbo et al., 1993, 2000; Maeda et al., 1995; Shahaf and Marom, 2001; Corner et al., 2002; Marom and Shahaf, 2002; van Pelt et al., 2004; Eytan and Marom, 2006; Wagenaar et al., 2006; Ham et al., 2008; Stegenga et al., 2008). Here we implement a previously described response-clamp method (Wallach et al., 2011) to control the responsiveness of *in vitro* cortical networks to external stimulation over many hours, and point at several constraints and tradeoffs entailed by externally enforced stability. We show that network response probability and response latency, significantly fluctuating under open loop conditions, may be effectively restrained and manipulated by the closed loop algorithm. This ability relies on the fact of monotonic relations between stimulation amplitude and both response probability and latency.

We find that over time, enforced response stability leads to activity dependent dispersion of pair-wise correlations between times to first-spikes across the network, as well as destabilization of global input–output relations. These, we interpret, reflect reduced efficacy of internal homeostatic processes (reviewed in, e.g., Turrigiano and Nelson, 2004; Davis, 2006; Marder and Goaillard, 2006; Turrigiano, 2008, 2011; Maffei and Fontanini, 2009; Marder and Tang, 2010), which appear to be compromised by our externally imposed closed loop drive. Stated differently, under open loop conditions, the network responds when it can; where it cannot, no response is evoked and resources are regained. However, under closed loop conditions, when the network is incapable of responding to a given input, the amplitude of the latter is further increased, enforcing use of whichever activity resources are available; these, in turn might give rise to the observed plastic changes. This does not mean that open loop stimulation cannot induce plasticity; there are many reports that demonstrate this (e.g., Jimbo et al., 1998, 1999; Madhavan et al., 2006; Chao et al., 2007; Chiappalone et al., 2008; Vajda et al., 2008; Bologna et al., 2010; le Feber et al., 2010). We also observe activity dependent dispersion of pair-wise correlations between first-spikes across the network, under open loop stimulation. Enforced stable, high levels of activity by closed loop conditions seem to accelerate such changes. This interpretation is congruent with that offered by le Feber et al. (2010), describing enhanced changes in connectivity measures upon implementation of adaptive (closed loop) stimulation compared to open loop.

The extent to which the proposed *in vitro* experimental toy model offered here prove useful for the study of closed loop conditions *in vivo*, remains to be seen.

## 4. Materials and methods

### 4.1. Cell preparation

Cortical neurons were obtained from newborn rats (Sprague–Dawley) within 24 h after birth using mechanical and enzymatic procedures described in earlier studies (Marom and Shahaf, 2002). Rats were killed by CO_2_ inhalation according to protocols approved by the Technion's ethics committee. The neurons were plated directly onto substrate-integrated multi electrode arrays and allowed to develop into functionally and structurally mature networks over a period of 2–3 weeks. The number of plated neurons was of the order of 450,000, covering an area of about 380 mm^2^. The preparations were bathed in MEM supplemented with heat-inactivated horse serum (5%), glutamine (0.5 mM), glucose (20 mM), and gentamycin (10 μg/ml), and maintained in an atmosphere of 37°C, 5% CO_2_, and 95% air in an incubator as well as during the recording phases. An array of Ti/Au extracellular electrodes, 30 μm in diameter, spaced 500 μm from each other (MultiChannelSystems, Reutlingen, Germany) was used. The insulation layer (silicon nitride) was pre-treated with polyethyleneimine (Sigma, 0.01% in 0.1 M Borate buffer solution).

### 4.2. Electrophysiology

A commercial amplifier (MEA-1060-inv-BC, MCS, Reutlingen, Germany) with frequency limits of 150–3000 Hz and a gain of ×1024 was used for obtaining data. Data was digitized using an acquisition board (PD2-MF-64-3M/12H, UEI, Walpole, MA, USA). Each channel was sampled at a frequency of 16 kHz. Action potentials were detected on-line by threshold crossing. The thresholds (8 × root mean square units; typically in the range of 10–20 μV) were defined separately for each of the recording channels before the beginning of the experiment. All spike times and voltage traces were recorded for analyses. Electrical activity detected often originates from several sources, typically 2–3 neurons, as each recording electrode is surrounded by several cell bodies. Detection of NSs was performed on-line by threshold crossing of spike rate (20 action potentials recorded throughout the electrode array within a 25 ms time bin), considering a refractory period of about 500 ms. Voltage stimulation was applied in a monophasic 200 μs square pulse 100–1000 mV, through extracellular electrodes, using a dedicated stimulus generator (MCS, Reutlingen, Germany). The electrode chosen for stimulation was the one showing the highest probability to participate first in spontaneous NSs, typically assuring the capability to evoke reliable network responses. Using Simulink software, processing and analyzing of data on-line was paced at 500 μs iteration steps [see Zrenner et al. (2010) for more details]. Data acquired was further analyzed off-line using Matlab (Mathworks, Natick, MA, USA).

### 4.3. PI control

Proportional-Integral (PI) controller was realized on the xPC target system (Levine, 1996). The input to the controller is the error signal,

(1) $$e_{n}=P^{*}(n)-P_{\tau }(n)$$

where P^*^(*n*) and *P*_τ_(*n*) are the desired and estimated response probabilities at the *n*th stimulus, respectively. The output of the controller is generally composed of three components,

(2) $$A_{n}=A_{baseline}+g_{P}e_{n}+g_{I}\sum_{i=1}^{n}e_{i}$$

where *g*_*P*_ and *g*_*I*_ are the proportional and integral gain parameters, respectively. Here, we typically used *g*_*P*_ = 1 (400 mV) and *g*_*I*_ = 0.2 (80 mV), and *A*_*baseline*_ was the baseline amplitude bias (set according to an open loop stimulation amplitude required for reaching the desired reference response probability).

On-line estimation of network response probability: Let *y*(*n*) be an indicator function, so that *y*(*n*) = 1 if the network generated a NS within a predefined interval after the *n*th stimulus (10–800 ms), and *y*(*n*) = 0 otherwise. We denote *P*_τ_(*n*) the estimation calculated at time *t* > *t*_*n*_, based on the set of responses {*y*(1), *y*(2),…, *y*(*n*)} to stimuli given at times {*t*_1_, *t*_2_,…, *t*_*n*_}. A weighted average was realized by using the recursive formula,

(3) $$P_{\tau }(n)=(1-e^{-\frac{t_{n}-t_{n-1}}{\tau }})\cdot y(n)+e^{-\frac{t_{n}-t_{n-1}}{\tau }}\cdot P_{\tau }(n-1)$$

The estimation time-constant, τ, was typically set to 250 s.

## Funding

The research leading to these results has received funding from the European Union Seventh Framework Programme (FP7/2007-2013) under Grant agreement No. FP7-269459 CORONET, and was also supported by a grant from the Ministry of Science and Technology, Israel, and funding organization of EU countries.

### Conflict of interest statement

The authors declare that the research was conducted in the absence of any commercial or financial relationships that could be construed as a potential conflict of interest.
